# Learning curve for laparoscopic radical prostatectomy

**DOI:** 10.20452/wiitm.2025.17933

**Published:** 2025-02-10

**Authors:** Anna Barnaś, Tomasz Milecki, Agnieszka Ida, Michał Kasperczak, Adam Lipski, Andrzej Antczak, Wojciech A. Cieślikowski

**Affiliations:** Department of Urology, Poznan University of Medical Sciences, Poznań, Poland; Department of Urology, Józef Struś Multispecialist Municipal Hospital, Poznań, Poland

**Keywords:** laparoscopy, learning curve, prostate cancer, prostatectomy

## Abstract

**INTRODUCTION:**

While robotic prostatectomies are gaining popularity, laparoscopic and open surgeries remain prevalent in Central and Eastern Europe due to their cost‑effectiveness. In Poland, many urology residents report insufficient laparoscopic training. This study retrospectively evaluated the learning curve for laparoscopic radical prostatectomy (LRP).

**AIM:**

This paper aimed to assess the learning curve of a single resident performing extraperitoneal LRP.

**MATERIALS AND METHODS:**

We analyzed 72 patients who underwent LRP between 2016 and 2020 at a single center, divided into 5 groups (G1–G5) according to chronological order. The procedures were per‑ formed by a single urologist without on‑site supervision. Outcomes included operative duration, length of hospital stay, complications, transfusion rates, histopathology findings, biochemical recurrence, and urinary incontinence.

**RESULTS:**

Patient characteristics were similar across all groups. The median (interquartile range [IQR]) age ranged from 61 (54–66) to 68 (66–70) years, and the median (IQR) prostate‑specific antigen con‑ centration, from 6.7 (5.4–8.5) to 15 (6.3–19.3) ng/ml. Higher Gleason scores were more common in the G3 and G4 groups (P = 0.05) than in the other groups. Surgery time decreased from 183 minutes in the G1 group to 130 minutes in the G5 group (P <0.001). The rates of positive surgical margins were the highest in the G3 and G4 groups (53.3% and 46.7%, respectively; P = 0.02). The rate of urinary continence improved from 66.7% in the G1 group to 86.7% in the G4 group (P = 0.36); however, without any significant difference among all groups. Biochemical recurrence rates tended to be lower in the G4 and G5 groups (6.7% and 8.3%, respectively), but the difference across all groups was nonsignificant. Grade III–V complications occurred only in the G1 group.

**CONCLUSIONS:**

Surgical outcomes improved after 15 procedures, and the oncological outcomes, after 45, with functional improvement observed later. Performing hundreds of surgeries may be required to achieve high proficiency in performing LRP.

## INTRODUCTION

Robotic prostatectomies are becoming standard worldwide. However, in Central and Eastern Europe, laparoscopic or open surgeries remain common treatment methods in daily clinical practice. These techniques are still being used as they are non inferior in terms of oncological outcomes and cost-effectiveness.^1^

The laparoscopic technique is widely accessible in most training centers in Poland. Nevertheless, many urology residents perceive their proficiency in this technique as unsatisfactory.

Furthermore, a considerable number of urology residents lack access to comprehensive training opportunities.^2^ Notably, the surgical expo- sure among Polish urology residents predominantly involves minor laparoscopic procedures.^3^ Laparoscopic technique learning is associated with intra- and postoperative complications, but their rate decreases with the duration of training. Later, in comparison with the initial stages of training, the percentages of leakages and positive margins decrease, blood transfusions are less often needed, the operative and hospitalization times are shortened, and biochemical recurrence (BCR) occurs less often.^4^

**TABLE 1:** Patient characteristics

Parameter		Study group					P value
		G1 (n=15)	G2 (n=15)	G3 (n=15)	G4 (n=15)	G5 (n=12)	
Age, y		61 (54–66)	61 (57–67)	65 (59–70)	65 (58–69)	68 (66–70)	0.11
BMI, kg/m²		27.4 (27.9–30)	27.7 (25.2–29)	27.8 (26.6–32.1)	29.4 (26.6–32.7)	27.6 (26.1–28.7)	0.83
PSA, ng/ml		6.7 (5.4–8.5)	7.4 (7–12)	15 (6.3–19.3)	8.3 (7.1–17.1)	8.2 (7.1–13.5)	0.13
Prostate volume, ml		30 (25–47.5)	30 (22.5–37.5)	30 (30–40)	35 (30–44.5)	30 (20–48.3)	0.67
Preoperative Gleason score	3 + 3	11 (73.3)	11 (73.3)	8 (53.3)	7 (46.7)	10 (83.3)	0.09
	3 + 4	4 (26.7)	2 (13.3)	5 (33.3)	1 (6.7)	1 (8.3)	
	4 + 3	0	1 (6.7)	1 (6.7)	5 (33.3)	0	
	4 + 4	0	1 (6.7)	0	2 (13.3)	1 (8.3)	
	4 + 5	0	0	1 (6.7)	0	0	

Data are presented as median (interquartile range) or number (percentage).

Abbreviations: BMI, body mass index; PSA, prostate‑specific antigen

**TABLE 2:** Postoperative results

Parameter	Study group					P value
	G1 (n=15)	G2 (n=15)	G3 (n=15)	G4 (n=15)	G5 (n=12)	
Operative duration, min, mean (SD)	183 (38.4)	152 (28.2)	167 (35.1)	157 (29.1)	129 (17.1)	<0.001
Rectal injury	2 (13.3)	0	0	0	0	–
Transfusion	1 (6.7)	0	0	0	0	–
Anastomotic leakage	1 (6.7)	0	0	0	0	–
Hospital stay, d	4 (3.5–6)	4 (4–6)	5 (4–6)	4 (4–6)	4 (3.8–7.3)	0.94
Urinary retention	0	0	0	0	0	–
Continenceᵃ	10 (66.7)	13 (86.7%)	9 (60%)	13 (86.7%)	9 (75%)	0.36
Clavien–Dindo grade III complication	3 (20)	0	0	0	0	–
BCR	4 (26.7)	3 (20%)	4 (26.7%)	1 (6.7%)	1 (8.3%)	0.08

Data are presented as number (percentage) or median (interquartile range) unless indicated otherwise.

a Using ≤1 pad per day.

Abbreviations: BCR, biochemical recurrence

Even today, the learning curve for laparoscopic prostate surgery remains poorly defined. The knowledge of the learning curve is essential for a urology trainee. It improves patient safety during operations, recovery, and oncological outcomes, and may affect the operator’s motivation. Therefore, we retrospectively analyzed prostatectomy cases at a single center to determine the learning curve of laparoscopic radical prostatectomy (LRP).^5^

## AIM 

The objective of this study was to evaluate the learning curve associated with extraperitoneal LRP performed by a single resident.

## MATERIALS AND METHODS 

### Study cohort and data sources 

We analyzed 72 patients who were hospitalized in the years 2016–2020 and underwent LRP in a tertiary center. The participants were al located to 5 groups (G1–G5) according to chronological order: the groups G1–G4 included 15 patients each, and the G5 group comprised 12 patients. The inclusion criteria were as follows: men with biopsy proven prostate cancer, treatment naive, willing to undergo radical prostatectomy, with a life expectancy greater than 10 years. Patients with bone metastases, comorbidities that represent contraindications to laparoscopic surgery, and bleeding disorders were excluded from the study. The study patients declined other treatment options, such as radiotherapy with or without hormonal therapy or active surveillance. Before surgery, all of them had a consultation with an anesthesiologist and received premedication.

### Procedure description

All prostatectomies were performed by a single urologist during his residency and after completing his specialty training. At that time, there was no on site specialist experienced in laparoscopy in the center; the specialist was available only by phone. All patients were previously diagnosed with localized prostate adenocarcinoma. Physical therapists helped with patient mobilization on the first day after surgery. All patients were discharged with a temporary bladder catheter in place, which was typically removed 10 to 14 days after surgery. Complications were evaluated according to the Clavien–Dindo classification.^6^

We retrospectively assessed the duration of the operation, length of hospital stay after LRP, transfusion rates, intra- and postoperative complications (eg, rectal injury), histopathologic results, and BCR. BCR was defined as a prostate- -specific antigen (PSA) level greater than or equal to 0.2 ng/ml confirmed by measurement 6 weeks after surgery. The surgery time was counted from the first incision to the placement of the final suture. The length of hospital stay was defined as the number of days from the day of surgery to discharge. Incontinence was assessed by the need to use more than 1 pad per day. Data on patient demographics, surgical details, and outcomes were obtained from hospital medical records, and included operative reports, histopathologic findings, and discharge summaries. Tumor staging was performed according to the TNM classification (eighth edition),^7^ where T represents the size and extent of the primary tumor, N indicates the absence or presence of regional lymph node metastases, and M refers to the absence or presence of distant metastases. Path- ological staging (pT) was based on histopathologic findings after radical prostatectomy. We did not evaluate sexual function, as an inadequate number of patients had the International Index of Erectile Dysfunction assessed be- fore the procedure and we could not compare the results before and after surgery. Moreover, most patients were not in the low-risk prostate cancer group; thus, nerve-sparing techniques were not used. The average follow-up time was 2 years; the patients were monitored for BCR.

**FIGURE 1:**
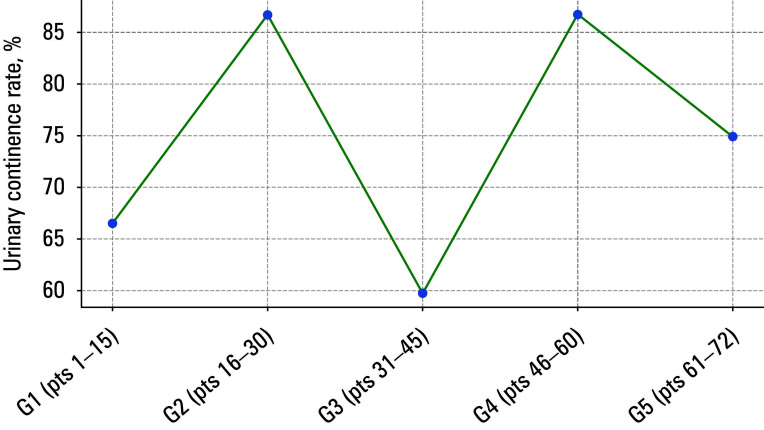
Locally weighted scatter plot smoothing curve for urinary continence

Abbreviations: G, group; pts, patients

**FIGURE 2:**
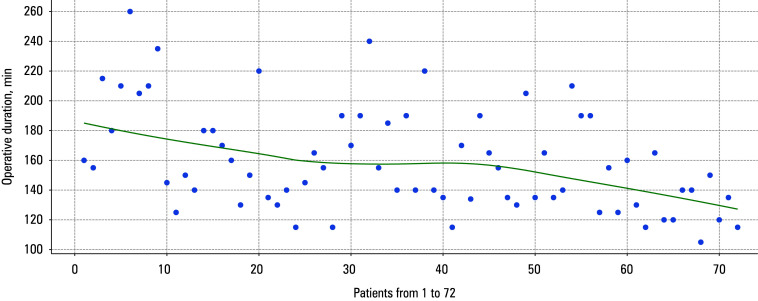
Locally weighted scatter plot smoothing curve for operative duration

### Statistical analysis

Statistical analysis was performed using the SPSS software, version 25.0 (IBM Corp., Armonk, New York, United States). Numerical variables with a normal distribution were reported as mean (SD), and non-normally distributed variables, as median (interquartile range [IQR]). Categorical variables were presented as number (percentage). The analysis of variance (ANOVA) test was used for continuous variables. The Kruskall–Wallis test was used when the assumptions of the parametric ANOVA test were not met. The χ^2^ or Fisher exact test was used for nominal variables. A P value below 0.05 was considered significant.

### Ethics

This retrospective study did not require a bioethics committee approval. Patient data were anonymized to ensure confidentiality, and all procedures followed ethical guidelines for retrospective studies.

## RESULTS

Patient characteristics are presented in TABLE 1. No significant differences among the 5 study groups were observed in that do- main. The median (IQR) age ranged from 61 (54–66) to 68 (66–70) years, and the median (IQR) PSA concentration, from 6.7 (5.4–8.5) to 15 (6.3–19.3) ng/ml. In the G3 and G4 groups, higher Gleason scores were more common, but this finding was nonsignificant.

The mean (SD) duration of surgery decreased in each subsequent group of patients (P <0.001; TABLE 2, starting from 183 (38.4) minutes in the G1 group and ending at 129 (17.1) minutes in the G5 group. However, no significant dif- ferences were observed in the length of hos- pital stay.

**FIGURE 3:**
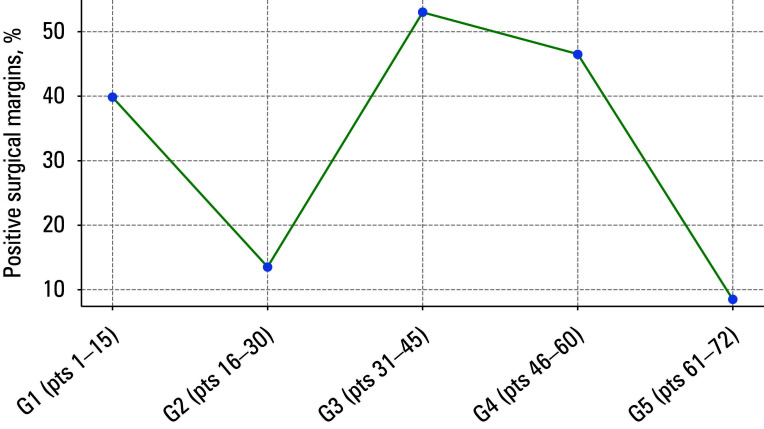
Locally weighted scatter plot smoothing curve for positive surgical margins

**FIGURE 4:**
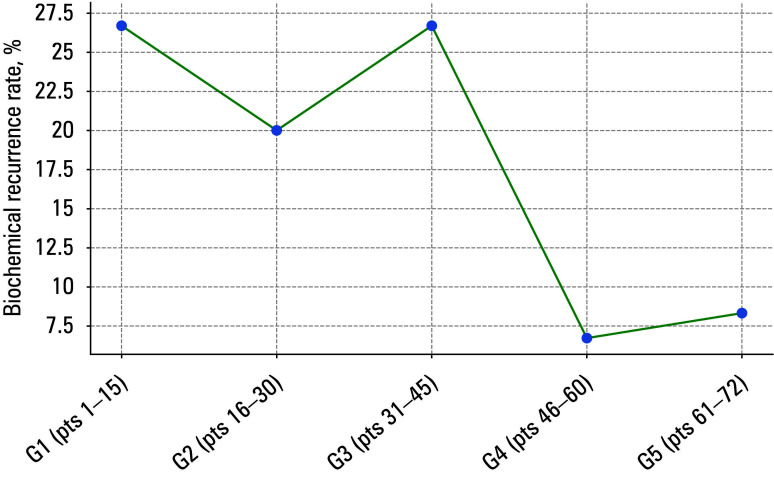
Locally weighted scatter plot smoothing curve for biochemical recurrence

Locally weighted scatter plot smoothing (LOWESS) curves for urinary continence, operative duration, BCR, and positive surgical margins (PSMs) are presented in FIGURES 1–4.

The PSM rates and histopathologic staging for each group are shown in TABLE 3. The groups differed in terms of margin positivity (P = 0.02) and histopathologic staging (P = 0.004). For these 2 variables, the results showed a sinusoid pattern rather than a specific trend. However, in each sub- sequent group of patients, there were more so-called challenging patients, that is, those with more locally advanced disease than in the initial group. In the G1 group, patients with pT3 dis- ease accounted for 53% of the cohort, but none of them had seminal vesicle invasion. At the same time, in the G3 group, the percentage of patients with pT3 disease was 66.7%. In the G4 group, the percentage of individuals with pT3 disease was 40%, but 83.3% of those patients had seminal vesicle invasion. The highest PSM rates were observed in the G3 and G4 groups (53.3% and 46.7%, respectively). These were the only groups that included patients with seminal vesicle invasion (pT3b), which significantly complicated maintaining a negative margin. Eventually, only a single patient in the G5 group had cancer cells at the surgical margin.

Similarly, results for urinary continence TABLE 2 followed a sinusoidal pattern FIGURE 1, with patients in the G4 and G5 groups preserving continence at rates of 86.7% and 75%, respective- ly, while in the G1 group, only 10 patients (66.7%) remained continent. The Fisher exact test showed no difference across all 5 groups (P = 0.36).

Biochemical recurrence rates TABLE 2;FIGURE 3 were lower in the G4 and G5 groups (6.7% and 8.3%, respectively) than in the G1 and G3 (26.7% each) and G2 (20%) groups. Having compared the G1–G3 groups with the G4 and G5 groups, we found no difference in the BCR rate (P = 0.08). Clavien–Dindo grade III complications TABLE 2, were observed in 3 patients from the G1 group (rectal injury in 2 individuals and anastomotic leakage in a single patient). Similarly, the need for blood transfusion occurred solely in a single patient in the G1 group.

## DISCUSSION

The learning curve for laparoscopic surgery represents a complex process of acquiring technical skills and achieving proficiency, of- ten influenced by the surgeon’s experience and case volume. To monitor progress effectively, it is essential to establish specific end points, such as operative time, blood loss,^8^ and complication rates, including the need for reintervention.^9^ In oncological procedures, factors such as negative surgical margins and recurrence rates also serve as critical indicators of surgical outcomes.

In our study, we evaluated surgical, functional, and oncological outcomes of patients undergoing LRP performed by a single urology resident. We analyzed 72 patients divided into 4 groups of 15 individuals and 1 group of 12 individuals. This allowed us to closely monitor the operator’s progress during subsequent surgeries and helped us identify when it occurred. To the best of our knowledge, no other study has utilized a similar grouping of patient cohorts.

**TABLE 3:** Positive surgical margins and histopathologic staging after laparoscopic radical prostatectomy

Histopathologic staging	Study group					Overall M+/ total (%)a	P value^b^
	G1	G2	G3	G4	G5		
	M+/total (%)a						
pT1	0/0	0/0	0/0	0/0	0/0	0/0	0.02
pT2a	0/0	0/2	0/2	0/3	0/1	0/8	
pT2b	0/0	0/1	0/1	1/1 (100)	0/1	1/4 (25)	
pT2c	0/7	1/6 (16.7)	1/2 (50)	1/5 (20)	0/6	3/26 (11.5)	
pT3a	6/8 (75)	1/6 (16.7)	4/6 (66.7)	1/1 (100)	1/4 (25)	13/25 (52)	
pT3b	0/0	0/0	3/4 (75)	4/5 (80)	0/0	7/9 (77.8)	
pT4	0/0	0/0	0/0	0/0	0/0	0/0	
Total	6/15 (40)	2/15 (13.3)	8/15 (53.3)	7/15 (46.7)	1/12 (8.3)	24/72 (33.3)	

Most importantly for patient well-being, onco- logical outcomes improved after the surgeon had operated on the first 45 individuals. With respect to the surgical margin assessment, the results may raise skepticism regarding progress. How- ever, as mentioned earlier, in each subsequent group of patients, there was a higher number of patients with locally advanced disease. This in- creases the level of surgical difficulty, which must be considered when assessing progress in surgery. A similar perspective needs to be adopted when analyzing continence in patients.

### Complications 

As mentioned above, the initial surgeries were associated with concerns about severe complications in patients. In our study, only 3 out of 72 patients (4.2%) experienced se- vere complications (grade III–V according to the Clavien–Dindo classification). Furthermore, those occurred only in the first 15 patients. Blood trans- fusion was needed in a single patient from the G1 group. In a similar study by Gregorio et al,^10^ severe complications occurred in only 1 out of 82 patients (1.2%), that is, even less frequently than in our study. That study compared the results of residents learning LRP with those of experienced operators. The transfusion rate was 9.7% and did not differ from that observed among the specialists (8.5%; P = 0.68).

In a study by Luke et al,^11^ rectal injury and complications of grade IIIa or higher according to the Clavien–Dindo classification occurred in 1.6% of patients, and the blood transfusion rate was 3.1%. That study was conducted on 207 patients divided into groups of 50 individuals each, with the last group consisting of 57 patients. A training case was identified when the fellow complet- ed at least 2 of the 10 steps of the procedure. All participating fellows had spent at least 12 months in the authors’ unit and had basic upper urinary tract laparoscopy skills but no prior experience with LRP. Before starting the fellowship, they were given access to dry laboratory laparoscopic training and video recordings of the primary au- thor’s operative technique. In our study, the trainee operator had prior experience with specific stages of laparoscopic surgeries, including LRP. We assumed that the procedure was performed independently when the operator completed all stages of surgery without assistance from another specialist.

In a study by Good et al,^12^ severe complications occurred in 2 patients out of the first 100 operat- ed, whereas in the entire cohort, they were pres- ent in 8 out of 550 patients (1.5%). Only 2 pa- tients required blood transfusion (0.36%).

The above findings suggest that fears of se- vere complications in patients are unfounded and should not hinder skill development.

### Positive surgical margin

Other crucial aspects for patients, apart from complications, include long-term oncological prognosis, assessment of recurrence risk, and potential need for adjuvant treat- ment.^13^ A PSM is one of the risk factors for disease progression and may be harder to avoid for an inexperienced operator. As time passes by and we acquire new skills, we progressively encounter more challenging cases than at the beginning of our journey. In patients with more advanced disease, maintaining negative margins is more difficult. Such a situation occurred in our study, as the groups G3 and G4 comprised patients with advanced disease (individuals with a histopathologic pT3 result comprised 66% of the G3 group and 40% of the G4 group). Our study groups differed in surgical margin positivity (P = 0.02) and histopathologic staging (P = 0.004).

In a study by Handmer et al,^14^ the disease was organ-confined (pT2) in 76% of the first 100 patients, and the PSM rate in that group was 18.4%. In the second 100 patients, pT2 disease was present in 71% of the operated men, and the PSM rate was 17.5%. No difference was found in margin positivity (P = 0.62). In the patient groups with pT3a and pT3b disease, the percentage of positive margins was 34.8% and 52.9%, respectively, for the first 100 operated patients. No significant difference was observed between the first and second 100 operated patients in those cases. In another previously mentioned study,^11^ in the LRP learning group, the percentage of positive margins in the patients with pT2 disease was 2.8%, whereas in the group of specialized operators, it was 15.3% (P = 0.05). However, in the case of patients with pT3 disease, the PSM rate was 52% for trainees and 45.1% for LRP specialists (P = 0.55).

According to a systematic review that included 17 studies on LRP,^5^ significantly lower PSM rates were achieved after 50–60 procedures, and a plateau was established at 150–350 surgeries.

The results described above are consistent with ours and demonstrate that improvements in oncological outcomes similar to those obtained by trained specialists can be achieved relatively quickly.

### Biochemical recurrence

We observed a lower BCR rate after operating on the first 45 patients, but the finding was nonsignificant. The BCR rate in the groups G1–G3 ranged from 20% to 26.7%, while in the groups G4 and G5, it was 6.7% and 8.3%, respectively. Similar findings were reported in a study by Gregorio et al,10 where the BCR rate in the patients with pT2 and pT3 disease who were operated on by residents was 6% and 9.7%, respectively—nearly twice as high as in the case of operations performed by specialists, who served as the comparative group. However, the authors indicated that the greater BCR rate among the patients operated on by the residents was probably due to longer follow-up (10 months vs 56.7 months). Sivaraman et al15 showed that BCR rates for the first 50, 50–350, and more than 350 LRP cases were 30%, 20%, and 16.7%, respectively (P <0.001). However, in that study, unlike in ours, the percentage distribution of patients with pT2 and pT3 disease in each group was not specified.

### Urinary continence

Finally, we evaluated patient functional outcomes. While some improvement in continence was observed among the study partic- ipants, no significant differences were noted be- tween the groups (P = 0.36). In a similar study by Gregorio et al,^10^ the global continence rate was 52.4% (43/82). However, the authors defined mild urinary incontinence as the use of 1 pad per day, whereas we considered patients in such cases as continent. Of note, the above results are highly unsatisfactory. The study by Good et al^12^ showed that continence plateaued after operating on approximately 250 patients, whereas a systematic review^5^ indicated this effect after 70–350 surgeries.^5^ Maintaining continence and low BCR rates in patients is therefore one of the most challenging goals to achieve during the learning process of LRP.

### Limitations

The limitations of our study include its retrospective nature and a relatively small number of patients. Furthermore, our study assessed only 1 operator. Another factor that may make the analysis of the learning curve difficult is the varying levels of complexity among individual surgeries and patients. In everyday practice, it is not always possible to perfectly match the stage of disease advancement and the skills of the training operator, especially when the initial biopsy findings are underestimated. Apart from that, we only considered surgeries performed entirely by the investigated operator from start to finish. At that time, there were no more experienced laparoscopic mentors in our institution; thus, all the operations were performed from start to finish solely by the investigated operator. In our analysis, we did not consider procedures in which the operator performed only a part of the surgery, which could have added value to his overall training. Similarly, we did not include information on simulator training outside the hospital setting.

## CONCLUSIONS

In our study, improvement in surgical outcomes was noticeable after the trainee operator performed procedures in 15 patients. Oncological outcomes improved after performing 45 surgeries, and functional outcomes at the latest stage. Achieving high proficiency in LRP may re-quire performing hundreds of surgeries.
